# Effects of Different Crop Types on Soil Microbial Community Structure and Assembly in the Cold Temperate Region of Northeast China

**DOI:** 10.3390/microorganisms13112488

**Published:** 2025-10-30

**Authors:** Wenmiao Pu, Rongze Luo, Kaiquan Zhang, Zhaorui Liu, Hong Wang, Xin Sui, Maihe Li

**Affiliations:** 1 Engineering Research Center of Agricultural Microbiology Technology, Ministry of Education & Heilongjiang Provincial Key Laboratory of Ecological Restoration and Resource Utilization for Cold Region & Key Laboratory of Microbiology, College of Heilongjiang Province & School of Life Sciences, Heilongjiang University, Harbin 150080, China; puwenmiao@163.com (W.P.); hellolrz@163.com (R.L.); 18704568533@163.com (K.Z.); 13206828833@163.com (Z.L.); 13163400129@163.com (H.W.); 2 Forest Dynamics, Swiss Federal Institute for Forest, Snow and Landscape Research, CH-8903 Birmensdorf, Switzerland; 3 Key Laboratory of Geographical Processes and Ecological Security in Changbai Mountains, Ministry of Education, School of Geographical Sciences, Northeast Normal University, Changchun 130024, China; 4 School of Life Science, Hebei University, Baoding 071002, China

**Keywords:** soil microorganisms, crop types, community structure and assembly

## Abstract

Soil microorganisms play a crucial role in maintaining soil functionality and ecological balance by participating in key processes such as organic matter decomposition, nutrient cycling, soil structure formation, and plant health support. High-throughput sequencing was utilized in this study to systematically investigate the influence of different crop types, maize (*Zea mays*), soybean (*Glycine max*), and *Eleutherococcus senticosus*, on the communities and assembly mechanisms of soil microorganisms in a cold-temperate agroecosystem. The results reveal that cultivation practices led to significant differences in soil chemical properties compared to fallow land (CK). Total carbon (TC), total nitrogen (TN), and available nitrogen (AN) were significantly lower in CK than in cultivated soils, with the highest values observed in maize treatments among all crop types (*p* < 0.05). Furthermore, the alpha diversity of bacteria in the maize and soybean treatments was significantly higher than that in CK, while there was no significant difference between the *Eleutherococcus senticosus* treatment and CK. However, no significant differences were observed in the ACE and Chao1 indices of the soil fungal communities across the four crop types. Beta diversity of bacterial and fungal communities exhibited significant variations under different crop cultivation practices. Specifically, compared with CK, the relative abundance of *Sphingomonas,* which contributes to the degradation of complex organic compounds, and *Gemmatimonas*, which plays a role in nitrogen cycling, significantly increased, whereas the relative abundance of *Clavaria*, a genus capable of decomposing recalcitrant lignin and cellulose, decreased. Analysis of community assemblies revealed that both bacterial and fungal communities were predominantly influenced by deterministic processes across all crop types. This finding provides a scientific basis for maintaining soil fertility in a targeted manner, precisely protecting crop health and optimizing agricultural management efficiently, thereby supporting sustainable agricultural practices. In conclusion, by examining microbial diversity and community dynamics across different crops, along with the underlying environmental factors, this study aims to enhance our understanding of plant–microbe interactions and provide insights for sustainable agricultural practices in cold-temperate regions.

## 1. Introduction

Soil is a fundamental component of the Earth’s ecosystems, providing essential ecological services [1]. It acts as a natural filter and reservoir for water and nutrients, serves as a growth medium for plants and heterotrophic organisms, and offers habitats for a vast array of life forms [2]. Soil microbial communities play a crucial role in these processes, particularly in the decomposition of organic matter [3]. As one of the most abundant and diverse groups on the planet, soil microorganisms, especially bacteria, dominate microbial biomass, accounting for approximately 70–90% of the total, with fungi following in second place [4]. These microbes are integral to nearly all ecological processes affecting soil health. They help maintain soil functionality in both natural and agricultural systems by contributing to soil structure formation, organic matter decomposition, toxin removal, and the cycling of carbon, nitrogen, phosphorus, and sulfur [5,6]. Microorganisms also play vital roles in suppressing plant diseases, promoting plant growth, and influencing vegetation changes [7]. Therefore, understanding how soil microbial communities respond to environmental changes is crucial for advancing sustainable agriculture and effective land management.

Plants have a significant influence on the composition of soil microbial communities. Numerous studies have shown that vegetation influences microorganisms through various mechanisms. Root exudates supply essential carbon sources that fuel microbial activity, while plant litter alters nutrient composition and availability in the rhizosphere, affecting microbial community structure and function [8,9]. Variations in life form, metabolic processes, and root architecture among plant species exert distinct selective pressures on both soil physicochemical properties and microbial communities. For example, legumes like soybean enhance soil nitrogen-cycling microorganisms [10,11]. In contrast, grasses like maize exude phenolic-rich compounds that may suppress pathogenic microbes or stimulate beneficial phosphate-solubilizing bacteria, leading to different microbial assembly patterns [12]. These varying growth strategies, such as the nitrogen-fixing ability of legumes and the allelopathic effects of herbaceous plants, underscore the complex and multifaceted nature of plant–microbe interactions in soil ecosystems [13,14].

Maize (*Zea mays*) and soybean (*Glycine max*), two of the world’s most widely cultivated and economically important crops, have been extensively studied for their impacts on soil microbial communities [15]. As an annual cereal with high nutrient demands, maize is often linked to declining soil bacterial diversity, particularly in monoculture systems, which can lead to the proliferation of pathogenic fungi. Its intensive cultivation practices, such as frequent soil tillage, disrupt soil structure and microbial habitats, further exacerbating microbial diversity loss [16]. In contrast, soybean positively influences soil health through their symbiotic relationship with *Bradyrhizobium* bacteria, facilitating biological nitrogen fixation [17]. This process enriches soil nitrogen levels, fosters the growth of nitrogen-transforming microorganisms, and enhances soil fertility and sustainability [18]. While the soil ecological impacts of maize and soybean are well-documented, the effects of *Eleutherococcus senticosus*, a perennial woody species with notable medicinal value and economic potential, remain largely unexplored. With its robust root system and a long life cycle, *Eleutherococcus senticosus* releases root exudates rich in bioactive secondary metabolites, including lignans and essential oils [19]. These compounds likely exert unique influences on soil microbial communities, distinct from those associated with annual crops. Unlike herbaceous annuals such as maize and soybean, *Eleutherococcus senticosus* exhibits different growth habits, metabolic inputs, and soil disturbance patterns, suggesting it may influence the soil microenvironment through alternative ecological pathways.

Despite growing interest in plant–microbe interactions, most studies have focused on individual crop systems or specific microbial taxa, often within intensively managed agricultural landscapes [16,20]. There is still a lack of comparative, field-based research examining how diverse plant types influence soil microbial communities under relatively undisturbed, ecologically representative conditions. The study took place in the Shuanghe Nature Reserve, located in Tahe County inside the Daxing’anling district of Heilongjiang Province, China. The site offers a uniquely favorable research environment: situated in a cold temperate continental monsoon climate zone, it combines a long history of agricultural activity with minimal industrial disturbance and a relatively enclosed landscape [20,21]. These characteristics create a unique blend of ecological stability and agricultural relevance, making it an ideal setting for studying plant-driven effects on soil microbial communities. The site’s longstanding cultivation history and environmental isolation allow for controlled comparisons across different crop types, free from the confounding influence of pollution or urban encroachment.

In this study, we selected three representative plant species, maize (*Zea mays*), soybean (*Glycine max*), and *Eleutherococcus senticosus* to represent distinct crop types, with a fallow field serving as the control (CK). Our objectives were to (1) characterize differences in soil microbial communities and their assembly processes across the various crop types; (2) identify the environmental factors driving microbial community dynamics under each crop type and (3) test the hypothesis that root exudates or secondary metabolites from different plants, particularly those with distinct root architectures like *Eleutherococcus senticosus*, influence the structure and assembly of soil microorganisms. This study aims to enhance our understanding of the intricate relationship among crops, soil, and soil microorganisms, while providing valuable insights for promoting sustainable land use and crop management in cold-temperate agroecosystems.

## 2. Materials and Methods

### 2.1. Study Area

The research took place in Tahe County, located in the Daxing’anling Prefecture of Heilongjiang Province, within the confines of the Shuanghe Nature Reserve (124°52′48″–125°32′03″ E, 52°54′25″–53°12′08″ N) [22]. The reserve is situated in a cold temperate continental monsoon climate zone, characterized by an average annual temperature of −4.3 °C [23] and extremes ranging from −45.8 °C in winter to 38 °C in summer. The average annual precipitation is 460 mm, primarily occurring during the warm and humid summer months [24]. The terrain is predominantly low-lying and flat.

The study area has been under cultivation for over two decades, forming a typical agroecosystem. Its relatively isolated location, with minimal industrial pollution and other anthropogenic disturbances, provides an ideal setting for investigating the specific effects of crop types on soil microbial communities in farmland. The crop fields have been managed under conventional local practices, including annual plowing, chemical fertilization, and pesticide application when necessary, following continuous cropping for several years. No crop rotation was practiced in the selected fields during this study. The CK soils received no fertilization, tillage, or irrigation. In contrast, the soils for the treatment were consistently amended before sowing with 150 kg/ha of urea, 80 kg/ha of superphosphate, and 60 kg/ha of potassium chloride.

### 2.2. Experimental Design and Sample Collection

**Figure 1:**
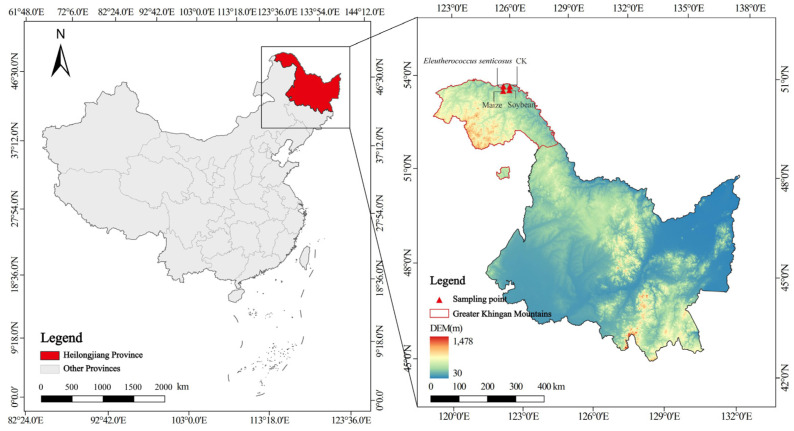
Geographical positions of the study. CK: fallow land; Soybean: *Glycine max*; Maize: *Zea mays*; *Eleutherococcus senticosus*.

This study employed a completely randomized block design to compare soil microbial communities under different land use types, represented by three common crops and a fallow control. The four treatments were: maize (*Zea mays*), soybean (*Glycine max*), *Eleutherococcus senticosus*, and fallow land (CK) (Figure 1). Each treatment had three independent replicate plots, resulting in a total of twelve experimental plots (10 m × 10 m each). To ensure spatial independence and minimize cross-contamination, replicate plots were spaced at least 20 m apart, and a 20 m-wide poplar windbreak was established between treatment blocks to prevent the exchange of irrigation water, soil, and microbes. Crop varieties and planting densities were selected in accordance with local agricultural practices.

Soil sampling was conducted in September 2024. This period was strategically chosen as the annual crops (maize and soybean) had reached physiological maturity, and the perennial *E. senticosus* was in its post-flowering stage, with all plants still actively growing in the field. This ensured that the soil microbial communities were under the active influence of living root exudates and rhizosphere processes, avoiding post-harvest disturbances. Furthermore, this time frame corresponds to a peak in soil nutrient cycling activity in this climate, ideal for capturing treatment-driven differences in microbial community structure and function. Sampling was performed on a sunny day to avoid confounding effects from recent precipitation.

With each plot, topsoil was collected from five points (four corners and the center) using a sterile soil auger, following a standard five-point sampling method [25]. After removing surface litter, the five subsamples from a single plot were composited into one bulk sample. The composite sample was homogenized by removing rocks and coarse roots on site, followed by thorough mixing in a sterile container for 5 min. The homogenized soil was immediately sealed in a sterile zip-lock bag, flash-frozen on dry ice, and transported to the laboratory within 8 h [26]. Upon arrival, each sample was subsequently divided into two aliquots under aseptic conditions: one was stored at −80 °C for molecular analysis (DNA extraction), and the other was air-dried at room temperature and passed through a 2 mm sieve for the analysis of soil physicochemical properties [27].

### 2.3. Determination of Soil Chemical Properties

Soil chemical properties were analyzed following standard procedures. Soil pH was measured in a 1:2.5 (*w*/*v*) soil-to-water suspension using a calibrated pH meter [28]. Soil total nitrogen (TN) and total carbon (TC) were determined by dry combustion at high temperature using an elemental analyzer (Elementar Vario EL III, Elementar, Langenselbold, Germany) [29]. Alkaline-hydrolyzable nitrogen (AN) was quantified using the alkaline diffusion method. In brief, the soil sample was treated with 1.0 M NaOH solution, and the released ammonia was absorbed in boric acid and subsequently titrated with standard HCl [30]. Available phosphorus (AP) was extracted with 0.5 M NaHCO_3_ (pH 8.5) and determined by the molybdenum-antimony colorimetric method. The phosphomolybdenum blue complex was developed by reduction with ascorbic acid, and absorbance was measured at 880 nm [31]. Available potassium (AK) was extracted with 1.0 M ammonium acetate extraction (NH_4_OAc, pH 7.0), and potassium concentration in the extract was determined using a flame photometer [32]. All measurements were conducted in triplicate, and mean values are reported.

### 2.4. DNA Extraction

Total genomic DNA was extracted from 0.5 g of frozen soil using the E.Z.N.A.^®^ Soil DNA Kit (Omega Bio-tek, Norcross, GA, USA) according to the manufacturer’s instructions. The concentration and purity of the extracted DNA were assessed using a NanoDrop spectrophotometer (Thermo Scientific, Wilmington, DE, USA), and its integrity was verified by 1% agarose gel electrophoresis.

The bacterial 16S rRNA gene (V3–V4 region) was amplified using the primers 338F (5′-ACTCCTACGGGAGGCAGCAG-3′) and 806R (5′-GGACTACHVGGGTWTCTAAT-3′) [33], while the fungal internal transcribed spacer (ITS1) region was amplified with the primers ITS1 (5′-CTTGGTCATTTAGAGGAAGTAA-3′) and ITS2 (5′-GCTGCGTTCTTCATCGATGC-3′) [34].

Polymerase chain reaction (PCR) was performed in a 20 μL reaction system comprising 0.5 μL of template DNA, 0.4 μL of each primer (10 μM), 4 μL of 5× TranStart^®^ FastPfu Buffer (TransGen Biotech Co., Ltd., Beijing, China), and 1 μL of 2.5 mM dNTPs, 0.4 μL of TransStart^®^ FastPfu DNA polymerase (2.5 U/μL), and nuclease-free water added to a final volume of 20 μL [35]. Thermal cycling conditions were as follows: initial denaturation at 94 °C for 5 min, 28 cycles of denaturation at 94 °C for 30 s, annealing at 55 °C for 30 s, and extension at 72 °C for 60 s; followed by a final extension at 72 °C for 7 min. For fungal amplification, the same conditions were applied except that 34 cycles were used [36]. Negative control (replacing DNA with nuclease-free water) was included in each PCR batch to detect potential contamination.

The PCR products were verified by 2% agarose gel electrophoresis and purified with AMPure XT Beads (Beckman Coulter Genomics, Danvers, MA, USA). The purified amplicons were quantified using a Qubit 3.0 fluorometer (Life Invitrogen, Thermo Fisher Scientific, Waltham, MA, USA). Following quantification, all samples were normalized to achieve uniform sequencing depth, pooled in equimolar ratios, and prepared for subsequent sequencing [37].

### 2.5. Bioinformatics and Statistical Analysis

High-throughput paired-end sequencing (PE300) was conducted on an Illumina platform (Illumina, San Diego, CA, USA) to profile both bacterial and fungal communities. Raw fastq reads were processed using QIIME 2 (version 2024.5). Primer sequences were removed with cutadapt (v5.0) [38] using the “--discard-untrimmed” option to ensure high-quality sequence retention. Quality control, chimera removal, and merging of paired-end reads were subsequently performed using the q2-dada2 plugin (DADA2 algorithm v1.36.0) [39].

For bacterial 16 S rRNA gene sequences, forward and reverse reads trimmed by 10 bp from the 5′ ends (--p-trim-left-f 10, --p-trim-left-r 10) to remove residual primers and low-quality bases. Based on quality score profiles, reads were truncated at 240 bp (forward) and 200 bp (reverse) (--p-trunc-len-f 240, --p-trunc-len-r 200). Sequences with a maximum expected error (--p-max-ee) greater than 2.0 were discarded. For fungal ITS sequences, parameters were set to --p-trim-left-f 0, --p-trim-left-r 0, --p-trunc-len-f 240, --p-trunc-len-r 200, with the same error threshold (--p-max-ee of 2.0). These steps yielded high-quality amplicon sequence variants (ASVs).

Taxonomic assignment was performed using the SILVA v138.2 reference database (accessed on 15 January 2025) and the UNITE v9.0 database (accessed on 15 January 2025) for fungal ASVs. The number of raw reads per sample ranged from 45,218 to 58,742 for bacteria and 42,891 to 56,103 for fungi, with post-filtering retention rates of 87.0% (38,954–50,129) and 86.1% (36,542–48,931), respectively. To standardize sequencing depth and minimize sampling bias, bacterial and fungal datasets were rarefied to 38,000 and 36,000 reads per sample, respectively. The resulting ASV tables were used for all subsequent analyses [40]. The raw sequencing data were deposited in the NCBI Sequence Read Archive (SRA) database under accession numbers: PRJNA1328057 (fungi), PRJNA1328056 (bacteria).

Differences in soil physicochemical properties among the four treatments were analyzed using one-way analysis of variance (ANOVA), followed by Duncan’s multiple range test for post hoc comparisons in SPSS (v 26.0; IBM Corp., Armonk, NY, USA). For microbial α-diversity indices (Chao1, ACE, Shannon, and Simpson), the non-parametric Kruskal–Wallis test was applied due to non-normality of residuals, followed by Dunn’s post hoc test with False Discovery Rate (FDR) correction in SPSS. Specifically, the Shannon index reflects community diversity by integrating species richness and evenness, whereas the Simpson index quantifies species dominance (with lower values indicating higher diversity/evenness). The Chao1 and ACE indices estimate species richness, with the former being more sensitive to rare taxa.

To assess β-diversity, principal coordinate analysis (PCoA) was conducted based on Bray–Curtis dissimilarity using the “vegan” package (v 2.6.6.1) [41]. Differences in community composition among treatments were tested using permutational multivariate analysis of variance (PERMANOVA) on the Bray–Curtis distance matrices. Genus-level abundance differences among crop types were tested using the Kruskal–Wallis test, and *p*-values adjusted for multiple comparisons using the false discovery rate (FDR) correction (*p* < 0.05). Redundancy analysis (RDA) based on Euclidean distance of ASV table was performed using the “microeco” package (v1.8.0) to explore the relationship between microbial community and soil physicochemical variables. Relationships among community composition, α diversity, and environmental variables were further evaluated using the Mantel test, with *p*-values adjusted by the Benjamini–Hochberg FDR correction to ensure reliable inference [42]. In the Mantel test, the Shannon index represents “diversity” and the ACE index represents “richness”, and Bray–Curtis dissimilarity represents “composition”. All statistical analyses were conducted in SPSS (version 26.0; SPSS Inc., Chicago, IL, USA) and R (v 4.4.1, R Foundation for Statistical Computing, Vienna, Austria, 2024), and all visualizations were generated in R.

## 3. Results

### 3.1. Soil Chemical Properties in Different Crop Types

Soil chemical characteristics varied significantly among crop types in the fallow land (CK) (Table 1; *p* < 0.05). The concentrations of TC, TN, and AN were highest in maize and lowest in CK. Conversely, AP and AK levels were highest in soybean and lowest in *Eleutherococcus senticosus*. The soil pH value was highest in CK and lowest in soybean.

### 3.2. Soil Microbial Diversity and Composition

In this study, the number of sequences obtained for each treatment group was as follows: soybeans had 58,643 bacterial sequences and 103,920 fungal sequences, with 5415 bacterial ASVs and 1230 fungal ASVs. CK had 67,060 bacterial sequences and 89,267 fungal sequences, with 4602 bacterial ASVs and 1328 fungal ASVs. *Eleutherococcus senticosus* had 57,205 bacterial sequences and 87,631 fungal sequences, with 5158 bacterial ASVs and 1686 fungal ASVs. Maize had 94,128.33 bacterial sequences and 85,894 fungal sequences, with 6123 bacterial ASVs and 1321 fungal ASVs. These results reflect the sequencing depth and microbial diversity within each treatment group.

**Figure 2:**
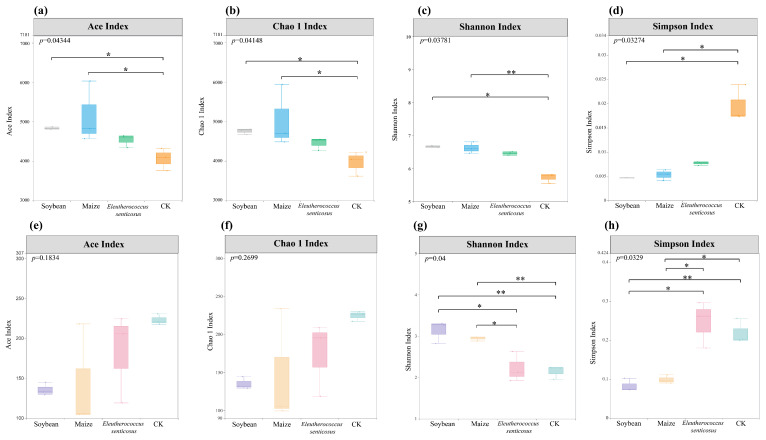
Boxplot showing bacterial and fungal α-diversity (Chao 1, ACE, Shannon, and Simpson indices) across different crop treatments. Subfigures: (**a**) bacterial Ace Index, (**b**) bacterial Chao1 Index, (**c**) bacterial Shannon Index, (**d**) bacterial Simpson Index, (**e**) fungal Ace Index, (**f**) fungal Chao 1 Index, (**g**) fungal Shannon Index, (**h**) fungal Simpson Index. Statistical differences were assessed using the Kruskal–Wallis test, followed by Dunn’s post hoc test with FDR correction. Significant differences between groups are indicated by star symbols (*, *p* < 0.05, **, *p* < 0.01). CK: fallow land (*n* = 3); Soybean: *Glycine max* (*n* = 3); Maize: *Zea mays* (*n* = 3); *Eleutherococcus senticosus* (*n* = 3).

Marked differences were found in the alpha diversity indices of soil bacterial communities across the crop types. The ACE and Chao 1 indices were significantly higher in the maize and soybean than in CK, while the Simpson index was significantly higher in CK. The Shannon index was significantly lower in CK compared to maize and soybean (Figure 2, *p* < 0.05). In contrast, no significant differences were found in the ACE and Chao 1 indices for soil fungal communities among the four crop types. However, the Simpson index was significantly higher in the *Eleutherococcus senticosus* and CK than in maize and soybean, whereas the Shannon index was significantly higher in soybean compared to *Eleutherococcus senticosus* and CK (Figure 2, *p* < 0.05).

**Figure 3:**
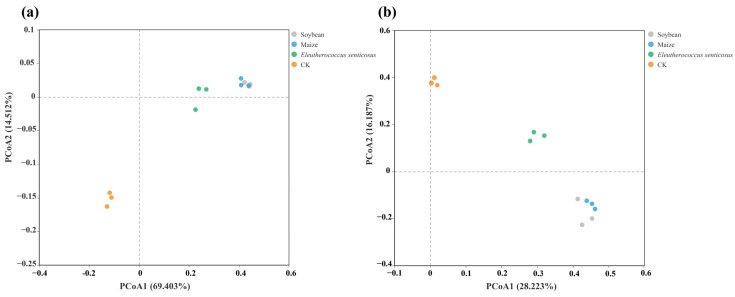
Principal Coordinate Analysis (PCoA) of soil bacterial (**a**) and fungal (**b**) communities based on Bray–Curtis dissimilarity. Each colored dot represents an individual sample from a specific crop treatment. CK: fallow land; Soybean: *Glycine max*; Maize: *Zea mays*; *Eleutherococcus senticosus*.

Principal Coordinates Analysis (PCoA) based on Bray–Curtis distances revealed clear differences in the β-diversity of soil bacterial and fungal communities among crop types (Figure 3, Tables S1 and S2). Variation in the soil bacterial community was primarily captured by the first and second principal coordinates (PCoA1: 69.4% and PCoA2:14.51%), which together accounted for 83.91% of the total variance (Figure 2a). For the fungal community, PCoA1 and PCoA2 accounted for 28.22% and 16.19% of the variance, respectively (Figure 3b).

**Figure 4:**
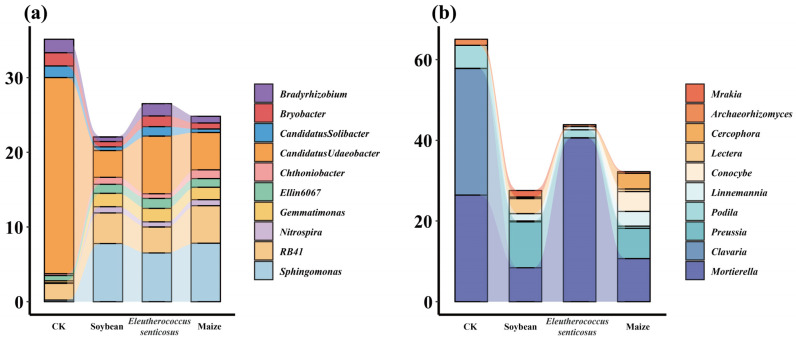
Relative abundance of dominant soil bacterial (**a**) and fungal (**b**) genera. Stacked bar charts illustrate the top ten genera across different crop treatments. CK: fallow land; Soybean: *Glycine max*; Maize: *Zea mays*; *Eleutherococcus senticosus*.

**Figure 5:**
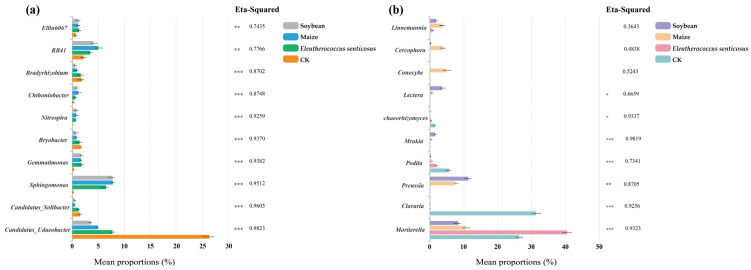
Relative abundances of bacterial (**a**) and fungal (**b**) genera under different crop treatments, determined using the Kruskal–Wallis test. The *x*-axis represents mean relative abundance (%) and the *y*-axis lists the genera. Asterisks indicate statistical significance: *p* < 0.05 (*), *p* < 0.01 (**), and *p* < 0.001 (***). Eta-Squared (η^2^) values represent the proportion of variance explained by crop type. CK: fallow land; Soybean: *Glycine max*; Maize: *Zea mays*; *Eleutherococcus senticosus*.

Crop cultivation also significantly altered the relative abundance of bacterial and fungal genera (Figure 4). For example, *Candidatus_Udaeobacter* and *RB41* were prominent among bacterial genera (Figure 3a), while *Mortierella* dominated the fungal community (Figure 4b). Compared to fallow land (CK), the relative abundances of *Sphingomonas* and *Gemmatimonas* significantly increased under crop cultivation (Figure 5a), whereas *Clavaria* declined (Figure 5b). LEfSe analysis identified 158 bacterial taxa as biomarkers for specific cropping systems (LDA > 4), including *Candidatus_Udaeobacter* in CK, *Sphingomonas* in *Eleutherococcus senticosus*, *RB41* in maize, and *norank_Gemmatimonadaceae* in soybean (Table S3, Figure S1a). Additionally, 192 fungal taxa were identified as biomarkers, including *Mortierella* in CK, *Linnemannia* and *Conocybe* in maize, *Mortierellaceae* in *Eleutherococcus senticosus*, and *Mrakia* and *Preussia* in soybean (Table S3, Figure S1b).

### 3.3. Soil Chemicals as Drivers of Soil Microbial Communities

**Figure 6:**
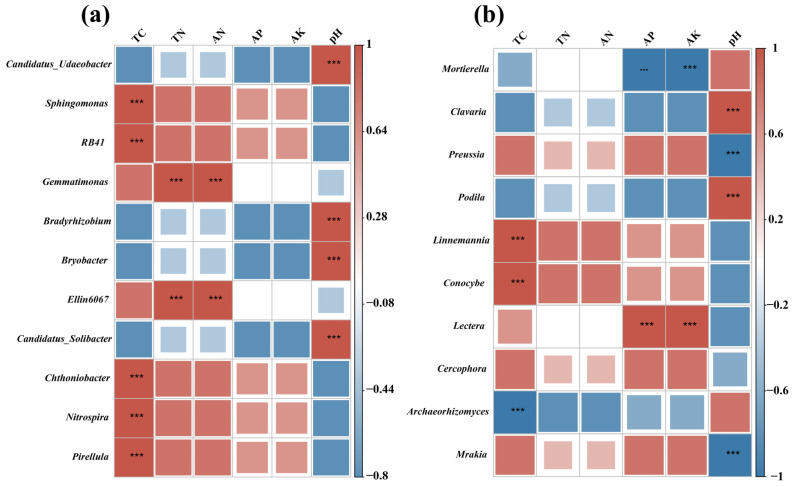
Heatmaps showing correlations between the ten dominant bacterial (**a**) and fungal (**b**) genera and soil chemical parameters. The colors Red and blue signify positive and negative correlations, respectively (*** *p* < 0.001). Correlations were calculated using Pearson’s correlation coefficients. TC: total carbon; TN: total nitrogen; AN: alkaline nitrogen; AP: Available P (Olsen); AK: Available K (NH_4_OAc).

Significant correlation was observed between the composition of soil microbial genera and soil physicochemical parameters. Specifically, bacterial genera such as *Sphingomonas*, *RB41*, *Chthoniobacter*, *Nitrospira,* and *Pirellula* showed positive correlation with TC (Figure 6a), while *Gemmatimonas* and *Ellin6067* correlated positively with TN and AN. Soil pH was significantly associated with *Candidatus_Udaeobacter*, *Bradyrhizobium*, *Bryobacter,* and *Candidatus_Solibacter*. At the fungal genus level (Figure 6b), *Linnemannia* and *Conocybe* correlated positively with TC, whereas *Archaeorhizomyces* showed a negative correlation. TC, AP, and AK were positively correlated with *Lectera* and negatively correlated with *Mortierella*. Soil pH showed strong positive correlations with *Clavaria* and *Podila*, and negative correlations with *Preussia* and *Mrakia*. It is important to emphasize that these correlations reflect statistical associations rather than confirmed causal relationships, as microbial communities and soil chemical properties may interact bidirectionally or be co-influenced by unmeasured factors.

**Figure 7:**
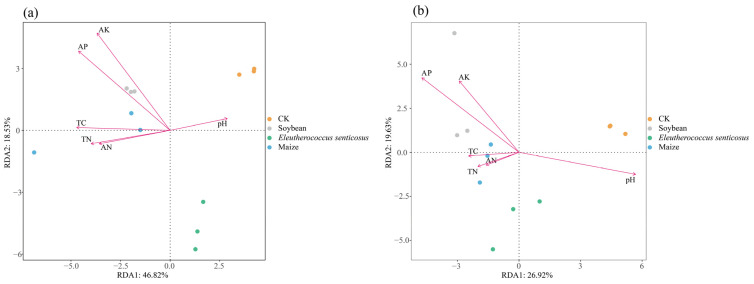
Redundancy analysis (RDA) of soil bacterial (**a**) and fungal (**b**) communities in relation to environmental variables across different crop types. Colors indicate different treatment groups. CK: fallow land; Soybean: Glycine max; Maize: Zea mays. TC: total carbon; TN: total nitrogen; AN: alkaline nitrogen; AP: Available P (Olsen); AK: Available K (NH_4_OAc).

Redundancy Analysis (RDA) was used to explore the relationships between soil microbial communities and environmental variables. TC, TN, and AN were positively correlated with bacterial community structure in the maize. AP and AK were positively linked to bacterial communities in soybean but showed negative associations in the *Eleutherococcus senticosus*. Soil pH showed a positive association with bacterial communities in the CK (Figure 7a). For fungal communities, TC, TN, and AN were negatively associated in the CK but positively associated in maize. AP and AK were positively associated with the fungal communities in the soybean, whereas soil pH exhibited a negative correlation (Figure 7b).

**Figure 8:**
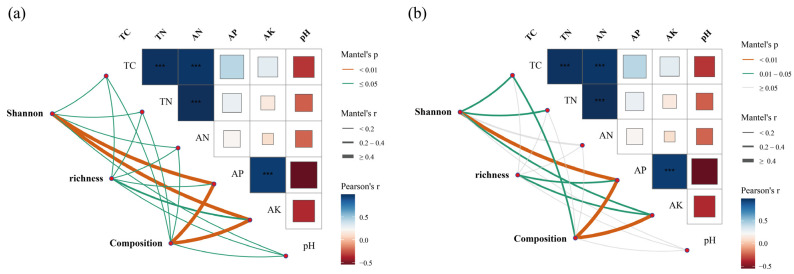
Mantel analysis showing relationships between soil bacterial (**a**) and fungal (**b**) community compositions, α-diversities, and soil chemical parameters (*** *p* < 0.001). Line color indicates Mantel’s *p* value, and line width indicates Mantel’s *r*. TC: total carbon; TN: total nitrogen; AN: alkaline nitrogen; AP: Available P (Olsen); AK: Available K (NH_4_OAc).

The Mantel test revealed significant associations between microbial community structure and key soil chemical parameters, particularly AP and AK (Figure 8). Bacterial Shannon diversity was strongly linked to AP and AK (Figure 8a), while fungal Shannon diversity correlated with AP (Figure 8b).

### 3.4. The Assembly Mechanisms of Soil Microbial Communities

**Figure 9:**
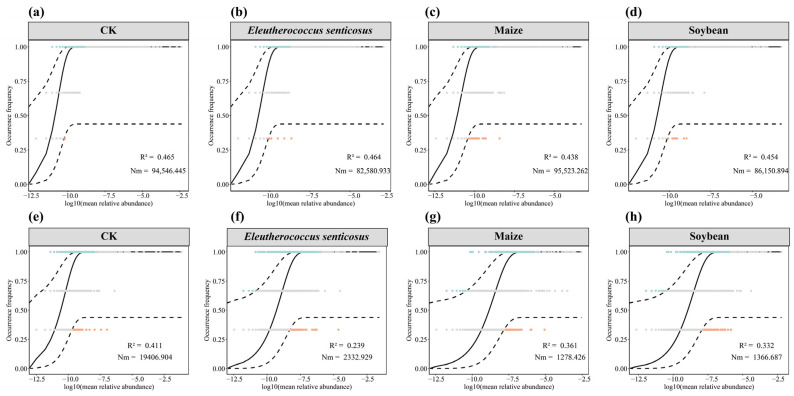
Fit of Neutral community model (NCM) for microbial community assembly. Predicted occurrence of bacterial (**a**–**d**) and fungal (**e**–**h**) communities across crop treatments. Panels (**a**–**d**) correspond to bacterial communities under CK, *Eleutherococcus senticosus*, Maize, and Soybean treatments, respectively; panel (**e**–**h**) shows the corresponding fungal communities. The solid black line indicates the best NCM fit, with dashed lines representing the 95% confidence intervals. Each point represents an Amplicon Sequence Variant (ASV); points within the confidence interval (gray) are considered neutrally distributed, points above the prediction (blue) and below the prediction (orange) deviate from neutral expectations. The coefficient of determination (R^2^) indicates model fit, and *Nm* (*Nm* = N × m) reflects the product of metacommunity size (N) and immigration rate (m), representing the overall dispersal rate within the community.

The assembly processes of bacterial and fungal communities under different crop types were assessed using the Sloan neutral community model. (Figure 8). Cultivation of different crops significantly affected microbial community assembly, altered the relative contributions of deterministic and stochastic processes. For bacterial communities (Figure 9a–d), deterministic processes constituted the predominant assembly mechanism. Notably, the maize treatment exhibited the highest dispersal capacity among the bacterial communities (*Nm* = 95,523). In contrast, crop cultivation led to reduced R^2^ values compared to the CK treatment, indicating a greater influence of deterministic processes. (Figure 9e–h).

## 4. Discussion

### 4.1. Variations in Soil Bacterial Community Structure Across Different Crop Types

Our findings demonstrate that different crop types exerted distinct influences on soil microbial α-diversity, with bacteria and fungi showing contrasting patterns. For bacteria, maize-planted soils exhibited significantly higher ACE and Chao 1 indices compared to other treatments (Figure 2), indicating enhanced bacterial richness. This enrichment is likely driven by maize root exudates, which are rich in organic acids, amino acids, and other low-molecular-weight compounds that serve as a readily available carbon source for soil bacteria, promoting their growth and increasing overall diversity [43].

Compared to the CK treatment, bacterial Shannon diversity was significantly higher under all three crop types, although no significant differences were observed among the crop types (Figure 2). This finding aligns with Fu [44], who reported that crop cultivation generally enhances soil bacterial diversity relative to uncultivated soils. The increase is likely attributable to root exudates and organic matter inputs, which create favorable conditions for microbial growth and stimulate a variety of bacterial taxa [45,46]. Practically, the pronounced enrichment of bacterial diversity under maize suggests that incorporating maize into crop rotation systems could enhance soil microbial diversity and function, supporting sustainable soil management.

In contrast, fungal richness, as indicated by ACE and Chao1 indices, did not differ significantly among the four land use types. However, soils under soybean exhibited the highest fungal Shannon diversity, significantly exceeding that of CK and *E*. *senticosus*. This result corroborates Song (2022), who reported that continuous soybean cultivation enhances fungal diversity [47]. Soybean roots release substantial amounts of flavonoids and phenolic acids, which can selectively shape fungal community composition [20]. These findings suggest that crops such as soybean, which promote fungal diversity, can be strategically utilized to improve soil health and ecosystem stability.

Principal Coordinates Analysis (PCoA) based on Bray–Curtis distances revealed that different crop types significantly impact β-diversity in both bacterial and fungal communities (Figure 3). Microbial compositions in maize and soybean soils were more similar to each other, likely because both herbaceous crops release small organic acids, such as citric acid and coumaric acid, which may foster similar microbial communities. In contrast, *E*. *senticosus*, a perennial medicinal shrub, releases distinct root exudates including terpenoids (e.g., eleutherosides) and pheolics, likely driving the development of a microbial community that is clearly differentiated from those of maize and soybean soils.

These findings highlight that crop-specific root exudate profiles are key drivers of soil microbial community structure [48]. From an agricultural management perspective, integrating annual crops such as maize and soybean with perennial medicinal species like *E. senticosus* in diversified rotations or intercropping systems could foster more complex and stable soil microbiomes. Such diversification may enhance critical ecosystem functions, including nutrient cycling and organic matter decomposition. Moreover, aligning crop-specific management with tailored fertilization strategies could further optimize microbial-mediated soil processes, thereby improving soil fertility and supporting the long-term sustainability of agroecosystems.

### 4.2. The Relationship Between Soil Microbial Communities and Soil Properties

Our results indicate that crop types significantly influence the relative abundance of soil bacterial and fungal genera (Figure 4 and Figure 5), likely reflecting differences in root exudate profiles. For example, *Sphingomonas* and *Gemmatimonas*, both commonly found in plant rhizospheres, were notably enriched under cropping systems. *Sphingomonas*, widely distributed in soils and on plant surfaces, exhibits broad metabolic capabilities that enable the degradation of diverse organic compounds, thereby contributing to organic matter decomposition and nitrogen and phosphorus cycling [49,50]. It also produces siderophores and indole-3-acetic acid (IAA), enhancing plant nutrient uptake (particularly iron) and promoting plant health [51]. *Gemmatimonas,* more abundant in agricultural soils, plays a key role in the nitrogen cycle, especially through N_2_O reduction, thereby improving nitrogen use efficiency [52,53]. These findings suggest that agricultural management practices can steer soil microbial communities toward specific functional traits. For example, integrating crops that release organic acids (e.g., maize) with nitrogen-fixing legumes (e.g., soybean) in rotation may simultaneously enhance carbon and nitrogen cycling, ultimately improving soil fertility.

LEfSe analysis identified several crop-specific microbial biomarkers (Figure S1). *Candidatus_Udaeobacter*, a biomarker for CK, is an oligotrophic bacterium adapted to nutrient-poor conditions and dominating in the absence of plant-derived inputs [54]. The enrichment of *Sphingomonas* in *E*. *senticosus* soil suggests that this medicinal plant selectively promotes beneficial bacteria through root exudates that supply essential nutrients and growth-promoting compounds [55]. Similarly, the prevalence of *RB41* in maize and *norank_Gemmatimonadaceae* in soybean underscores how crop-specific root exudates and nutrient dynamics shape microbial assembly [56,57,58]. Integrating a perennial medicinal shrub like *E*. *senticosus* into annual cropping systems may thus introduce unique microbial functional guilds, enhancing both functional diversity and soil microbiome resilience. The fungal community also exhibited shifts across crop types. *Mortierella* was more abundant under medicinal plant cultivation, consistent with its adaptation to nutrient-limited environments and oligotrophic lifestyle [59]. In soybean soils, *Preussia* and *Mrakia* were more enriched, likely due to favorable conditions resulting from nitrogen fixation and organic inputs [60,61]. These contrasting ecological strategies underscore the potential of crop diversification to maintain a wider spectrum of microbial functions, essential for ecosystem stability.

Strong correlations were observed between microbial communities and soil properties (Figure 6 and Figure 7). Among bacteria, *Sphingomonas* and *Chthoniobacter* showed positive correlations with total carbon (TC), aligning with their ability to degrade aromatic compounds and cellulose, thereby facilitating organic matter turnover [62,63,64,65]. *Gemmatimonas* correlated positively with TN and AN, reflecting its involvement in nitrogen cycling via the *nos*Z gene, N_2_O reduction, and phosphate accumulation [52]. These relationships support integrated soil fertility management, increasing soil organic carbon through cover crops or organic amendments fosters decomposition and carbon sequestration, while including nitrogen-fixing legumes like soybean enhances nitrogen availability and microbial taxa that optimize nitrogen use and mitigate N_2_O emissions. For fungi, *Linnemannia* and *Conocybella* correlated positively with TC, consistent with their roles in organic matter decomposition and carbon cycling [66,67]. Conversely, *Mortierella* was negatively correlated with AP and AK, reflecting its adaptation to low-nutrient conditions and its role as a saprotroph [68]. Its enrichment in *E*. *senticosus* soil suggests a preference for phosphorus- and potassium-limited environments, whereas higher nutrient levels favor competing fungal taxa.

The Mantel analysis highlights the pivotal role of soil phosphorus and potassium availability in shaping microbial community assembly (Figure 8). Microbial taxa respond sensitively to variations in these nutrients, which influence their metabolic activity, competitive interactions, and overall communities [69]. Phosphorus is an essential element for microbial energy transfer and nucleic acid synthesis, and its availability can determine microbial growth efficiency and community composition. Likewise, potassium regulates osmotic balance and enzymatic activity, indirectly affecting microbial physiological performance [70]. Collectively, these results imply that crop-induced changes in soil phosphorus and potassium dynamics could substantially reshape microbial diversity patterns, highlighting the importance of balanced nutrient management in sustaining soil microbial function and ecosystem stability [71].

### 4.3. Assembly Processes of Soil Microbial Communities Under Different Crop Types

The assembly of soil microbial communities is shaped by a combination of stochastic and deterministic processes. To assess their relative contributions, we applied the Sloan Neutra Community Model. (NCM) [72]. The model fit (R^2^) for both bacterial and fungal communities indicates that deterministic forces played a dominant role in shaping community assembly (Figure 9). These forces include biotic interactions, such as competition and antagonism, as well as abiotic filtering mediated by habitat conditions.

Notable differences in microbial dispersal (*Nm* values) were observed across treatments: Maize > CK > Soybean > *E*. *senticosus*. The higher *Nm* observed in maize soils may be attributed to its deep root system, which forms interconnected biopores that facilitate bacterial dispersal, potentially enhanced by surfactants in root exudates that improve microbial motility [73,74]. In contrast, *E*. *senticosus* soils exhibited the lowest *Nm*, suggesting restricted microbial dispersal. This could result from antimicrobial compounds in root exudates that inhibit bacterial activity, or from the secretion of sticky compounds that reduce soil porosity and impede movement [75].

Fungal community assembly was also strongly influenced by crop-specific factors. Tillage practices and root exudates, such as terpenoids released by *E. senticosus*, may act as selective agents, inhibiting mycelial growth and promoting deterministic assembly through both allelopathic and physical mechanisms [76,77].

While the NCM highlights the predominant role of deterministic factors in microbial community assembly, it alone cannot fully disentangle the contributions of assembly processes. Future research should incorporate complementary approaches, such as null model analyses (e.g., βNTI and RC), to more rigorously partition these components. Therefore, our interpretations remain cautious, and future studies are warranted to provide deeper insights into the complex interplay between deterministic and stochastic processes driving microbial community assembly.

### 4.4. Limitations and Future Prospects

This study has several limitations inherent to its small-sample, single-site design. While our results provide insights into how specific crop types influence soil microbial communities within the studied system, the generalizability of these findings may be limited by the restricted number of sampling sites and the unique environmental conditions at the study location. The small sample size per treatment reduces statistical power to detect subtle effects, and the single geographic context limits extrapolation to other soil types, climates, or management regimes.

To address these limitations and advance the field, future studies should adopt multi-site, large-scale sampling designs across varied soil types and ecological gradients. Sampling should also extend into subsoil layers (>20 cm) and incorporate long-term monitoring to assess microbial community resilience and delayed responses to surface disturbances. Additionally, investigating different agricultural practices, such as continuous cropping, intercropping, crop rotation, and intensive farming, will be critical for understanding their effects on microbial dynamics and informing sustainable soil management strategies.

The integration of quantitative methods, such as qPCR, is also recommended to complement amplicon sequencing data. This would enable high-throughput quantification of microbial abundance and functional genes related to key processes, such as nutrient cycling and pathogen suppression, providing a more mechanistic understanding of microbial responses to agricultural and environmental changes.

## 5. Conclusions

In conclusion, our research conducted in the Shuanghe Nature Reserve in Northeast China reveals how different crop types shape soil microbial diversity, composition, and assembly processes. Cultivation of *Eleutherococcus senticosus*, maize, and soybean significantly altered microbial communities compared with fallow soils. Maize enhanced bacterial diversity, whereas fungal richness (reflected by ACE and Chao1 indices) declined across all crop treatments. Soil chemical properties, such as total carbon, nitrogen, and available phosphorus, played a crucial role in shaping microbial community structure, with clear genus-level correlations to nutrient availability. Community assembly was governed largely by deterministic processes, with maize soils exhibiting the highest bacterial dispersal capacity. These findings highlight the complex interactions between soil properties and microbial communities, emphasizing the ecological implications of crop selection and the differential roles of deterministic and stochastic processes in microbial community assembly. This study deepens our understanding of the relationship between crops, soil, and soil microorganisms—specifically, the distinct effects of maize, soybean, and *Eleutherococcus senticosus* on soil microbial diversity and community structure. Beyond providing a scientific basis for sustainable land use planning and crop management in cold-temperate agroecosystems, the findings also offer actionable insights for optimizing sustainable agricultural practices: for instance, given that maize-enriched bacterial diversity and soybean-fostered rhizosphere microbial interactions showed complementary advantages, a maize–soybean rotation system could be recommended in this region to leverage their synergistic effects on soil microbial function—promoting balanced nutrient cycling while mitigating the decline in fungal richness observed under continuous single-crop cultivation. Furthermore, by identifying key drivers of microbial community structure, the results offer practical insights for optimizing agricultural practices to promote soil health and long-term ecosystem resilience.

## Figures and Tables

**Table 1:** Soil Chemical properties under different crop types.

Crop Types	TC (g/kg)	TN (g/kg)	AN (mg/kg)	AP (mg/kg)	AK (mg/kg)	pH
CK	15.5 ± 0.25 d	1.08 ± 0.06 c	116.2 ± 0.58 d	11.87 ± 0.58 c	12.38 ± 0.58 b	6.39 ± 0.12 a
Soybean	26.37 ± 0.57 b	1.53 ± 0.03 b	146.39 ± 1.15 c	34.98 ± 1.15 a	18.42 ± 1.15 a	5.98 ± 0.06 b
*Eleutherococcus senticosus*	20.81 ± 0.57 c	1.61 ± 0.02 b	168.36 ± 2.32 b	6.79 ± 0.11 d	8.79 ± 0.12 c	6.16 ± 0.08 ab
Maize	52.07 ± 0.38 a	3.57 ± 0.05 a	384.35 ± 1.73 a	23.81 ± 0.58 b	14.42 ± 0.58 b	6.12 ± 0.11 ab

Note: Values are presented as mean ± standard error (*n* = 3). Different letters indicated statistically significant differences among treatments (*p* < 0.05, ANOVA followed by Duncan’s multiple range test). CK: fallow land; Soybean: *Glycine max*; Maize: *Zea mays*; TC: total carbon; TN: total nitrogen; AN: alkaline nitrogen; AP: Available P (Olsen); AK: Available K (NH_4_OAc).

## Data Availability

The original contributions presented in this study are included in the article/Supplementary Material. Further inquiries can be directed to the corresponding authors.

## Supplementary Materials

The following supporting information can be downloaded at https://www.mdpi.com/article/10.3390/microorganisms13112488/s1, Table S1: PERMANOVA analysis of bacterial community composition across different crop types. Table S2: PERMANOVA analysis of fungal community composition across different crop types. Table S3: Representative biomarkers (LDA > 4) identified by LEfSe analysis. Figure S1: LEfSe analysis of ASVs among the different crop types (CK, Soybean, Maize and *Eleutherococcus senticosus*). The circles represent taxonomic levels from Kingdom to Genus, radiating from inside to outside. Nodes in different colors indicate microbial taxa significantly enriched in the corresponding group and contributing to intergroup differences. Yellow nodes represent taxa that are not significantly different among the groups or have no significant effect on intergroup differences.
