# Supplementary material for: Effects of Different Crop Types on Soil Microbial Community Structure and Assembly in the Cold Temperate Region of Northeast China
Source: Microorganisms. 2025 Oct 30;13(11):2488. doi: 10.3390/microorganisms13112488 (PMC12654503; doi:10.3390/microorganisms13112488)
Supplement: Supplementary file 1 [file microorganisms-13-02488-s001.zip › microorganisms-3834595-supplementary.pdf]

Table S1 PERMANOVA analysis of bacterial community composition across different crop types

pairs	R^2	*p*-value	*p*-adjusted
Soybean vs Medicine	0.8313006	0.003	0.0045
Soybean vs CK	0.936641	0.003	0.0045
Soybean vs Maize	0.3027762	0.005	0.006
Medicine vs CK	0.9083629	0.001	0.003
Medicine vs Maize	0.6544229	0.006	0.006
CK vs Maize	0.8306259	0.001	0.003

CK: fallow land; Soybean: *Glycine max*; Maize: *Zea mays*; Medicine: *Eleutherococcus senticosus*.

Table S2 PERMANOVA analysis of fungal community composition across different crop types

pairs	R^2	*p*-value	*p*-adjusted
Soybean vs Medicine	0.5479422	0.002	0.003
Soybean vs CK	0.7316826	0.001	0.003
Soybean vs Maize	0.3224468	0.003	0.0036
Medicine vs CK	0.7151466	0.001	0.003
Medicine vs Maize	0.6123289	0.002	0.003
CK vs Maize	0.7591678	0.004	0.004

CK: fallow land; Soybean: *Glycine max*; Maize: *Zea mays*; Medicine: *Eleutherococcus senticosus*.

Table S3 Representative biomarkers (LDA > 4) identified by LEfSe analysis

Crop types	Bacterial biomarkers	Fungal biomarkers
CK	*Candidatus_Udaeobacter*	*Mortierella*
Soybeans	*norank_Gemmatimonadaceae*	*Mrakia, Preussia*
Medicine	*Sphingomonas*	*Mortierellaceae*
Maize	*RB41*	*Linnemannia, Conocybe*

CK: fallow land; Soybean: *Glycine max*; Maize: *Zea mays*; Medicine: *Eleutherococcus senticosus*.

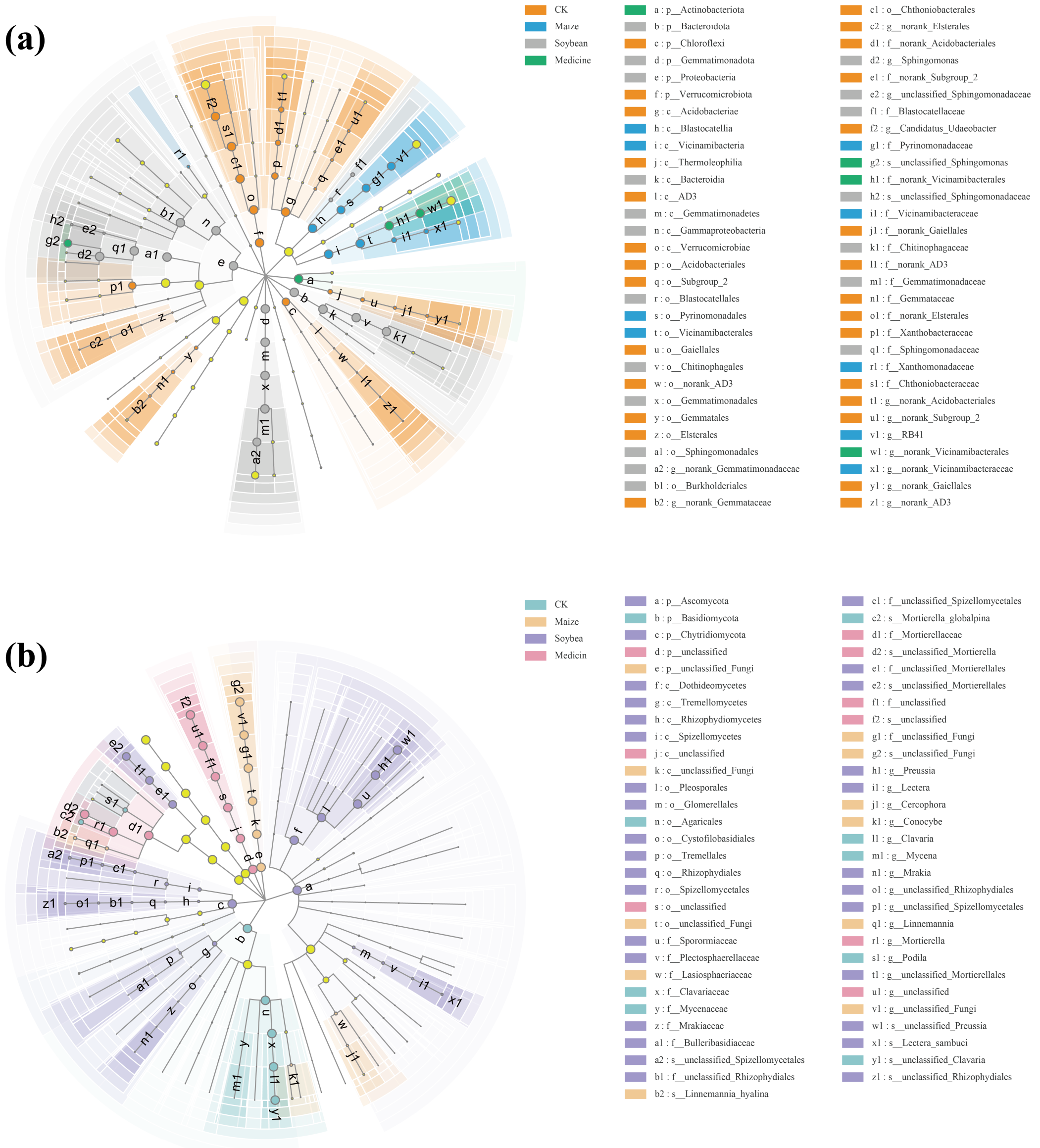

Figure S1 LEfSe analysis of OTUs among the different crop types (CK, Soybean, Maize and Medicine). The circles represent taxonomic levels from Kingdom to Genus, radiating from inside to outside. Nodes in different colors indicate microbial taxa significantly enriched in the corresponding group and contributing to intergroup differences. Yellow nodes represent taxa that are not significantly different among the groups or have no significant effect on intergroup differences.
